# Assessing Function and Endurance in Adults with Spinal and Bulbar Muscular Atrophy: Validity of the Adult Myopathy Assessment Tool

**DOI:** 10.1155/2014/873872

**Published:** 2014-05-05

**Authors:** Michael O. Harris-Love, Lindsay Fernandez-Rhodes, Galen Joe, Joseph A. Shrader, Angela Kokkinis, Alison La Pean Kirschner, Sungyoung Auh, Cheunju Chen, Li Li, Ellen Levy, Todd E. Davenport, Nicholas A. Di Prospero, Kenneth H. Fischbeck

**Affiliations:** ^1^Research Service/Geriatrics and Extended Care, Washington, DC Veterans Affairs Medical Center, 50 Irving Street, NW, Room 11G, Washington, DC 20422, USA; ^2^School of Public Health and Health Services, George Washington University, 2033 K Street, NW, Suite 210, Washington, DC 20006, USA; ^3^Rehabilitation Medicine Department, Clinical Center, Department of Health and Human Services (DHHS), National Institutes of Health (NIH), 10 Center Drive, Bethesda, MD 20892, USA; ^4^National Institute of Neurological Disorders and Stroke (NINDS), Neurogenetics Branch, Department of Health and Human Services (DHHS), National Institutes of Health (NIH), Building 35, Room 2A-1000, 35 Convent Drive, MSC 3705, Bethesda, MD 20892, USA; ^5^Department of Epidemiology, University of North Carolina at Chapel Hill Gillings, School of Global Public Health, 170 Rosenau Hall, Campus Box 7400, 135 Dauer Drive, Chapel Hill, NC 27599, USA; ^6^Center for Patient Care and Outcomes Research, Medical College of Wisconsin, 8701 Watertown Plank Road, Milwaukee, WI 53226, USA; ^7^Clinical Neurosciences Program, NINDS, NIH, 10 Center Drive, Room 5N230, Bethesda, MD 20814, USA; ^8^Neurology Department, University of Maryland, 110 South Paca Street, Baltimore, MD 21201, USA; ^9^Physical Medicine and Rehabilitation Service, Veterans Affairs Medical Center, 650 East Indian School Road, Phoenix AZ 85012, USA; ^10^Department of Physical Therapy, Thomas J. Long School of Pharmacy & Health Sciences, University of the Pacific, 3601 Pacific Avenue, Stockton, CA 95211, USA

## Abstract

*Purpose*. The adult myopathy assessment tool (AMAT) is a performance-based battery comprised of functional and endurance subscales that can be completed in approximately 30 minutes without the use of specialized equipment. The purpose of this study was to determine the construct validity and internal consistency of the AMAT with a sample of adults with spinal and bulbar muscular atrophy (SBMA). *Methods*. AMAT validity was assessed in 56-male participants with genetically confirmed SBMA (mean age, 53 ± 10 years). The participants completed the AMAT and assessments for disease status, strength, and functional status. *Results*. Lower AMAT scores were associated with longer disease duration (*r* = −0.29; *P* < 0.03) and lower serum androgen levels (*r* = 0.49–0.59; *P* < 0.001). The AMAT was significantly correlated with strength and functional status (*r* = 0.82–0.88; *P* < 0.001). The domains of the AMAT exhibited good internal consistency (Cronbach's **α** = 0.77–0.89; *P* < 0.001). *Conclusions*. The AMAT is a standardized, performance-based tool that may be used to assess functional limitations and muscle endurance. The AMAT has good internal consistency, and the construct validity of the AMAT is supported by its significant associations with hormonal, strength, and functional characteristics of adults with SBMA. This trial is registered with Clinicaltrials.gov identifier NCT00303446.

## 1. Introduction 

The adult myopathy assessment tool is a standardized, observed, physical performance test designed to be administered relatively quickly in clinical and research settings with common clinical equipment and minimal training (see Table 6 for the list of the AMAT tasks and scoring criteria). The AMAT consists of a 13-item battery with an ordinal grading scale for each item and a summated composite functional subscale (range = 0–21), endurance subscale (range = 0–24), and total score (range = 0–45), where lower AMAT subscale scores and total score indicate decreased physical performance. The functional and endurance domains that comprise the AMAT reflect the contribution of impaired muscle force on functional limitations [1–4] and incorporate recent findings that physical performance in people with and without myopathy are also affected by excessive fatigue [5, 6].

The AMAT items include common movements found in other field tests and clinical assessments [7–13], and have been adapted to feature integrated timed and criterion-based scoring within discrete measurement domains (i.e., functional and endurance AMAT subscales). In addition, the functional and endurance AMAT subscales are organized to be congruent with the disability models proposed by both the Institute of Medicine (IOM) [14] and the World Health Organization (WHO) [15]. The functional and endurance subscales were combined for the total AMAT score to imbue the assessment tool with important analytic advantages specifically in assessing patients with myopathy. A strict functional assessment battery based on the attainment of a transfer or mobility task may exhibit a significant ceiling effect (more than 15% of subjects attain the maximum score) if patients have muscle force above what is needed to complete the task for a single repetition. However, impairments in these individuals could be revealed during a more demanding endurance task. In contrast, an endurance battery may display a significant floor effect (more than 15% of subjects attain the minimum score) if patients do not have adequate muscle capacity to meet the criteria for a sustained or repetitive task [16]. Yet, these same individuals may demonstrate the requisite strength to complete a single repetition of a less demanding functional task. Integrating these high and low demand tasks into the AMAT total score diminishes the potential floor and ceiling effects of the assessment tool. Additionally, the AMAT items were sequenced to minimize the effects of fatigue by avoiding consecutive endurance tests of a given agonist muscle group. This assessment was also designed to have clinical utility. Therefore, it may be completed in 25–35 minutes and requires only common equipment such as a stopwatch, adjustable height examination table, standard stairs, and a goniometer. Moreover, the AMAT subscales and total score have been shown to have high interrater and intrarater reliability (ICC_2,1_ = 0.95–0.98, *P* < 0.0001) [17].

A sample of individuals with spinal and bulbar muscular atrophy (SBMA or Kennedy disease), an X-linked degenerative neuromuscular disorder caused by a CAG trinucleotide repeat expansion in the first exon of the androgen receptor gene (*AR*) [18], participated in this study. Briefly, SBMA is characterized by muscle fasciculations and cramping, bulbar weakness that may result in dysphagia and dysarthria [19, 20], and weakness of the proximal and distal muscles that often leads to impaired mobility and perceptions of excessive fatigue during upright mobility [19]. This sample was initially recruited for a larger clinical trial [21] and was used as a model of neuromuscular disease to help determine selected analytic properties of the AMAT.

There are few standardized scales available for the assessment of impairments and functional limitations due to SBMA [19, 25]. Furthermore, self-report assessment tools may not adequately capture observed functional performance or physical status [26–28]. The purpose of this study was to determine the construct validity of the AMAT for adult participants with SBMA disease. Secondary aims included determining the internal consistency of the AMAT domains and the relationship between functional AMAT subscale items and anatomic regional strength values. Our final aim was to determine if AMAT cut scores can be defined to reflect significant differences in strength, activities of daily living (ADL), timed 2 min walk, or self-reported physical status.

## 2. Methods

### 2.1. Participants

Fifty-six subjects (mean age, 53 ± 10 years) were recruited to the National Institutes of Health (NIH) Clinical Research Center in Bethesda, MD, for the purpose of participating in a trial to examine the efficacy and safety of dutasteride in SBMA [21, 29] (The trial is registered with Clinicaltrials.gov identifier NCT00303446); all data were obtained prospectively at the initial screening visit prior to the administration of dutasteride. Patient demographic information has been previously presented [29]. The study was approved by the National Institute of Neurological Disorders and Stroke Institutional Review Board. Signed photograph/recording release forms were obtained from healthy research volunteers in support of this project, and signed informed consent was obtained from study participants in accordance with the Declaration of Helsinki and Federal regulations. Inclusion criteria included: genetically confirmed SBMA, neurological symptoms of SBMA, ability to walk 100 feet with or without the use of an assistive device, male sex, and 18 years of age or older. Exclusion criteria included: female sex, less than 18 years of age, nonambulatory status, and any joint instability or other medical condition deemed by the investigators to pose an undue risk to participants engaging in the performance-based measures associated with the study.

### 2.2. Genetic Testing and Serum Androgen Profile

Blood samples were obtained after an overnight fast and processed in a CLIA-approved laboratory to assess androgen receptor gene CAG repeat length and serum androgen levels including total testosterone (TT), free testosterone (FT), and dihydrotestosterone (DHT).

### 2.3. Quantitative Muscle Strength Testing

**Figure 1:**
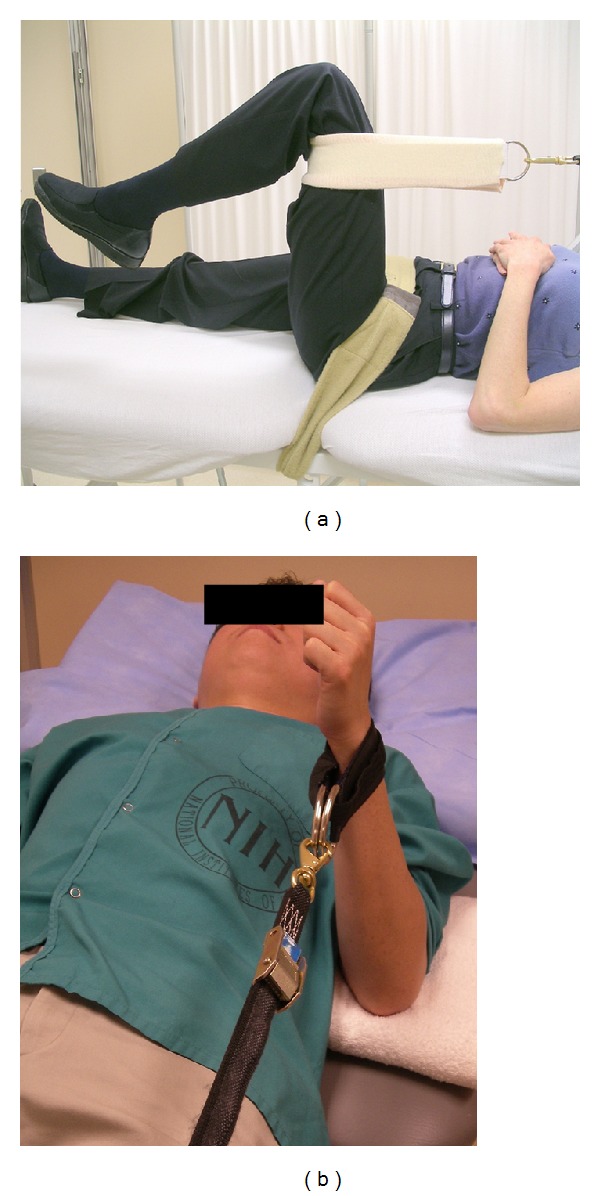
Isometric maximal voluntary contraction testing via the quantitative muscle assessment device. (Participant positioning shown for the: (a) hip extensors and (b) elbow flexors.)

Isometric maximal voluntary contraction (MVC) testing via quantitative muscle assessment (QMA) was used to measure peak force of bilateral muscle groups. The muscle groups and testing positions are listed in Table 7. All QMA tests were performed on a fixed-dynamometer (AEVERL Medical, LLC, P.O. Box 170, Gainesville, GA 30503) using load cells (Interface, 7401 East Butherus Drive, Scottsdale, AZ 85260) with computer-assisted data acquisition. The position of the strap (Figure 1) was adjusted to avoid contact with the participant and maintain a parallel orientation to the force vector. The dynamometer was calibrated per manufacturer guidelines and reset to “zero” prior to each MVC attempt to account for the passive force exerted against the strap. The mean value of the two MVC attempts was used for summation into a composite total score and anatomic region score (i.e., upper extremity and lower extremity).

### 2.4. Ambulation Status

Ambulation was assessed with the 2-minute walk test [25–30]. The timed 2-minute walk test has high reproducibility [30, 31] based on ICCs of 0.93. We administered 3 trials of the 2-minute walk test [32] as previously described [33], allowing for 2 practice trials before recording distance walked and gait speed. We compared the walk distance with the results of Selman and colleagues [24] to determine the predicted distance for age and gender matched controls.

### 2.5. Activities of Daily Living and Self-Reported Health Status

ADL assessment was modified [21, 29] from the ADL survey from the Friedreich ataxia rating scale (FARS) [34] by substituting a question about bladder control for one regarding difficulty with handwriting. While this questionnaire is validated for individuals with Friedreich ataxia [35], the ADL items reflect many of the limitations experienced by individuals with SBMA (i.e., walking, falling, swallowing, speech, dressing, personal hygiene, food handling and utensil use, and sitting position quality). The ADL assessment scores were inverted for statistical analysis, producing an ordinal 0–4 item scale (0 = maximum limitation; 4 = unaffected) and a summated composite total score of 36 (range = 0–36) with higher scores indicating increased levels of functioning. “Walking” and “falling” were individual ADL assessment items selected for additional analyses to better understand their relationship with AMAT performance.

Modules from the Medical Outcomes Study 36-item short form (version 2) questionnaire (SF-36v2) were used to obtain self-reported information on physical functioning and mental status. The SF-36v2 is a 36-item, 4-week recall health-related quality of life assessment that has been used in multiple disorders and can be condensed into 2 summary measures: the physical component summary (PCS) and the mental component summary (MCS) [36, 37]. Using the SAS code provided by QualityMetric Inc., raw scores were converted into normative-based scores with a mean score of 50 (standard deviation, ±10). The scoring algorithms for all SF-36v2 scales and summaries are gender- and age-matched and facilitate simple and valid comparisons between groups [38, 39].

### 2.6. Administration of the AMAT

A single physician with five years of experience with the AMAT administered the observed, physical performance test to the study participants. The test administrator issued instructions along with task demonstration for each AMAT activity before the participants attempted a given task. In addition, all participants were informed of the criteria to end each task (see Table 6) and the test administrator provided “standby” guarding to ensure participant safety during tasks requiring upright mobility. The participants were allowed a single attempt at completing each AMAT task; however, additional task attempts were allowed in the event of a procedural error during testing. The AMAT was initiated without warm up or preparatory activities and performed a minimum of 4 hours apart from the QMA and 2-minute walk test to avoid the negative impact of fatigue incurred from prior activity.

### 2.7. Data Analysis

Descriptive statistics were used to depict participant characteristics and all outcome measures. All data are expressed as means and standard deviations except individual AMAT item scores. The ordinal item scores are shown as median values and the interquartile range (IQR). Additionally, only the data distribution of the MCS and the functional subscale of the AMAT exhibited a significant departure from normality. Therefore, the data associated with these measures were the only variables requiring the use of nonparametric statistics [40]. In this study, the construct validity of the AMAT was based on the strength of its association with outcome measures that influence or reflect functional limitations and submaximal muscle endurance: androgen and genetic markers, muscle strength, timed 2-minute walk, ADL, and self-reported physical status. Construct validity is the extent that inferences may be made from the operational definitions within an assessment tool to the larger theory or concept of interest [40, 41]. Self-reported physical status, via the PCS, was expected to correlate with the AMAT and was used with the other outcome measures to assess construct validity. In contrast, self-reported mental status via the MCS was not expected to correlate with the AMAT and was used to establish divergent validity. Divergent validity of a given assessment tool is supported by a test outcome that lacks a significant association with variables presumed to measure different domains and should be independent of the outcome or construct of interest [41].

Pearson product-moment correlation coefficients (PMCC, *r*) and Spearman's correlation coefficients (Spearman's rho, *ρ*) were used to assess the association between variables, and the strength of the association among the variables was based on Munro's criteria [42]. Stepwise multiple linear regression analysis was used to determine the association between variables while accounting for the covariation among disease duration, CAG repeat length, TT, FT, and DHT [43]. All linear regression analyses and correlation coefficients involving QMA strength data included the values scaled to body weight (kg of MVC force/kg of body weight, resulting in a unitless value). This method of scaling strength data facilitated our analysis of the relationship between muscle strength and the functional tasks featured in the AMAT that involve the movement of body weight [44, 45]. QMA values were also expressed as a composite score (total QMA) and anatomic region scores (i.e., upper extremity and lower extremity QMA). Normative-based reference strength values, obtained from the National Isometric Muscle Strength (NIMS) Database Consortium [22] and Andrews and associates [23], were used for comparison with the SBMA group.

Low, moderate, and high levels of physical performance were determined by organizing subgroups of subjects based on cut scores derived from the AMAT total score tertiles. An analysis of variance (ANOVA) was used to discriminate among subjects with higher and lower levels of impairment [43]. The Kruskal Wallis test with Mann Whitney *U* post hoc tests were used for ADL falling and walking items since they involve ordinal data. Internal consistency of the functional and endurance AMAT subscales was assessed using Cronbach's alpha (*α*). These AMAT subscales represent related, but heterogeneous, aspects of physical functioning. Therefore internal consistency was evaluated for both AMAT subscales. Internal consistency is based on the pairwise correlations among the items within a subscale used to represent a given construct [40]. An* a priori* decision was made to consider Cronbach's *α* values of >0.70 as acceptable internal consistency of an AMAT subscale. In contrast, values exceeding 0.95 were considered indicative of a subscale with excessive item redundancy. Intra-item correlations were also calculated and coefficient values exceeding 0.85 indicated a redundant subscale item. The alpha level (two-tailed) was set at 0.05, and the statistical analyses were performed using SAS 9.1.3 (SAS Institute, Inc., Cary, NC), SUDAAN 9.0 for Windows (Research Triangle Institute Inc., Cary, NC), and SPSS statistical software version 10.0 for Windows (SPSS Inc., Street 233 S. Wacker Drive, Chicago, IL 60606).

## 3. Results

### 3.1. Participant Demographics and Disease Characteristics

The mean age of study sample at the time of trial participation was 53 (±10) years with a mean* AR* gene repeat length of 47 CAGs (range = 41–53). Detailed patient demographic information and serum androgen levels have been previously presented [29].

The participants with SBMA had diminished strength levels in comparison to the normative data. The MVC forces represented by the scaled total QMA score, scaled upper extremity (UE) QMA score, and scaled lower extremity (LE) QMA score were 42% to 65% of the reference values (Table 1). The mean distance travelled during the timed 2-minute walk was 109 ± 50 m for the participants corresponding to a mean velocity of 0.9 m/s (Table 1). Twenty-two of the 56 participants (39%) opted to use assistive devices (e.g., canes, walkers, or ankle-foot orthoses). These participants attained a mean distance of 66 ± 23 m with a mean speed of 0.55 m/s, whereas the individuals who did not use assistive devices achieved a mean distance of 136 ± 44 m (*n* = 34) with a mean speed of 1.13 m/s.

The ADL assessment score indicated that the participants experienced difficulties with physical functioning; the mean ADL assessment score was 25.9 ± 5.0 (range 15.0–35.3), representing 72% of the maximum attainable score. This is in agreement with the self-reported physical status in which the subjects had a mean PCS score of 34.3 ± 11.0 (16.0–57.8) which is 68% of the national age-matched normative data for men (35–74 years of age). In contrast, the self-reported mental status was noted by MCS mean scores of 52.2 ± 11.6 (14.2–67.2) which is 102% of normative values [38, 39].

### 3.2. The AMAT Subscale Scores and Total Score

Observed physical functioning, as measured with the AMAT, also revealed impaired performance of the participants. The mean total AMAT score was 29.2 ± 10.3 (i.e., 65% of the maximum AMAT total score) and no significant floor or ceiling effects were found in the AMAT total scores [16]. Of the 56 subjects, no one attained the low score of 0, and 2 participants achieved the maximum score of 45. In addition, slightly greater deficits were noted in the endurance AMAT subscale (60% of the maximum score) in comparison to the functional AMAT subscale (70% of the maximum score; Table 1). A range of performance ability was observed in both the functional and endurance AMAT subscales. Median item scores ranged from 1.0 to 3.0 for functional AMAT subscale items (item scale = 0–3) with the sit-up, sit to stand, and step-up tasks being the most difficult to perform. Median item scores varied across the full range of 0 to 4 for endurance AMAT subscale items (item scale = 0–4), with the repeated heel raises and repeated modified push-ups scoring the lowest (Table 2).

### 3.3. Outcome Variables Associated with the AMAT Total Score

The serum androgen levels had a moderate degree of association with the AMAT (*r* = 0.49–0.62; *P* < 0.001). The AMAT was significantly associated with CAG repeat length (*t* = −3.95; *P* < 0.001) when the multiple linear regression model corrected for age at evaluation and total testosterone as covariates. There was a stronger relationship between the AMAT and outcome measures related to physical performance. The total QMA score, timed 2-minute walk distance, and ADL assessment score all showed a high degree of association with the AMAT (*r* = 0.82–0.91; *P* < 0.0001). The self-reported physical status, as estimated by the PCS score, also correlated well with AMAT (*r* = 0.62; *P* < 0.0001) and, as hypothesized, the self-reported mental status via the MCS did not (*r* = 0.13; *P* = 0.355). Correlations between the AMAT total score and the outcome variables are summarized in Table 3.

### 3.4. Internal Consistency of the AMAT Subscales

The internal consistency of both AMAT subscales was acceptable based on the criteria established by Munro [42]. However, the internal consistency of the AMAT domains was stronger in the functional AMAT subscale (Cronbach's *α* = 0.89) than in the endurance AMAT subscale (Cronbach's *α* = 0.77). Intra-item associations of the AMAT subscales did not suggest item redundancy, as none of the correlation coefficients exceeded 0.85. The inter-item Spearman's *ρ* ranged from 0.39 to 0.74 for the functional AMAT subscale and 0.11 to 0.73 for the endurance AMAT subscale.

### 3.5. Strength-Function Relationships

Association between the functional AMAT subscale items and the QMA values was used to characterize strength-function relationships (Table 4). The total QMA, UE QMA, and LE QMA scores were significantly correlated with all of the functional tasks. The anatomic region QMA scores were more strongly associated with the functional tasks than the total QMA score, with the exception of the modified push-up. The UE QMA score had the highest degree of association with arm raise (*ρ* = 0.59; *P* < 0.001). In comparison, the LE QMA score had the highest degree of association with the supine to prone, sit-up, supine to sit, sit to stand, and the step-up tasks (*ρ* = 0.72–0.81; *P* < 0.001).

### 3.6. AMAT Cut Scores

Total AMAT score tertiles led to cut scores that separate the sample into low ≤ 24, moderate 25–34, and high ≥ 35 functioning groups. Significant differences were found among all 3 groups for the total QMA, timed 2-minute walk, total ADL, ADL falling, and ADL walking assessment scores (*P* < 0.001 for all main effects). Post hoc differences for ADL falling and walking were significant among all three groups; *P* < 0.001 in all comparisons except between the moderate and high functioning groups (*P* = 0.023). The low and high AMAT cut score groups showed significant differences in FT (*P* < 0.001), TT (*P* < 0.001), and DHT (*P* = 0.012), but not CAG repeat length (*P* = 0.41). In addition, the low and high and moderate and high AMAT cut score groups had significantly different physical status self-report scores (*P* < 0.001). All comparisons of the AMAT cut scores and outcome values in the functional domain are summarized in Table 5.

## 4. Discussion

### 4.1. Construct Validity of the AMAT

The findings of this investigation support the construct validity and internal consistency of the AMAT in participants with SBMA disease. Dependent measures obtained to characterize disease status and validate the AMAT included serum androgen levels,* AR* gene CAG trinucleotide repeat length, QMA scores, timed 2-minute walk, ADL assessment, and self-reported physical and mental status. Androgen levels are linked to the maintenance of muscle mass and strength [46], which in turn, leads to improved physical functioning [3, 47]. The relationship between the higher androgen levels and better functional performance was reflected in the significant correlation between the AMAT score and TT, FT, and DHT in the participants. We found a significant relationship between* AR* gene CAG trinucleotide repeat length and the AMAT total score, when accounting for the covariation of age at evaluation and TT. This finding supports other reports that CAG repeat length affects phenotypic measures of disease status [29, 48]. Additionally, previous work from our group [21] showed that there was an inverse correlation between CAG repeat length and QMA values scaled to body weight (*P* = 0.04).

The participants had significant impairment based on strength levels and walking distances that were approximately half of the normal adult reference values [24]. Also, the ADL assessment scores of the participants (25.9 ± 5.0; maximum attainable score = 36) were diminished, but similar to the clinical measures reported in other studies [49, 50]. The mean AMAT total score of 29.2 (±10.3; maximum attainable score = 45) reflects the decreased physical performance of the participants and is consistent with the findings regarding impaired muscle strength, ADL assessment, and self-reported physical status.

### 4.2. AMAT Subscale and Item Assessment

The AMAT subscales and items vary in their level of difficulty. Task difficulty is based on the proportion of body weight being moved and the distance traversed. However, task performance may be influenced by patterns of muscle weakness in people with neuromuscular disease. Based on the median item scores, supine to prone, modified push-up, supine to sit, and arm raise were the least demanding tasks of the functional AMAT subscale, while the sit-up, sit to stand, and step-up tasks posed the largest challenge to the participants. Sit to stand and ascending a step were expected to be challenging tasks due to the requirement to move one's total body weight and the reports of difficulty with these tasks in other cohorts. However, the data suggesting that the sit-up was the most difficult task was unexpected and has not been previously described in SBMA. Trunk weakness is a notable finding that has been observed in myopathies such as polymyositis and dermatomyositis [51]. Muscle groups of the extremities are typically more readily tested with dynamometry than trunk muscles, so the trunk musculature is typically omitted from objective strength assessment studies. Nevertheless, the observed difficulty with the sit-up task suggests that the trunk muscles may merit standardized objective strength assessment.

Sustained knee extension and hip flexion were the least difficult tasks of the endurance AMAT subscale, but even these tasks detected impairments in our sample (13 and 25 participants, resp., failed to reach the maximum score). Repeated heel raises and modified push-ups were clearly the most difficult tasks of the endurance AMAT subscale. The repeated heel raise task performance revealed the extent of distal weakness in the participants. The ankle plantar flexors can generate a large magnitude of force based on the lever type of the ankle joint and the muscle architecture of the gastrocnemius [52]. Despite these physiologic advantages, 39/56 subjects (70%) were unable to perform a single limb heel raise. The diminished performance of the participants for the repeated push-up task was of interest given the high scores attained on the single repetition version of this task in the functional AMAT subscale. The decreased performance of the repeated version of the push-up item may indicate sufficient strength to complete the task, but inadequate muscle endurance capacity to sustain task performance. Indeed, investigators have cited the need for endurance tests in addition to single repetition functional tasks alone to capture this important aspect of physical performance in persons with myopathy [6]. Repeated movements such as heel raises may be noted by performance deficiencies due to diminished strength and anaerobic capacity at ancillary muscle groups that contribute to stability during tasks with substantial multijoint involvement [53]. Additionally, SBMA is notable for being a lower motor neuron disease with significant muscle tissue abnormalities. Signs of significant muscle fiber damage such as elevated levels of serum creatine kinase often precede stereotypic SBMA clinical symptoms [54]. Also, muscle tissue in those with SBMA is distinguished by aberrant features such as fiber type grouping and centrally located nuclei which reflect characteristics of both neurogenic and myogenic pathology [55]. These morphological and histological abnormalities would contribute to the physical deficits observed in our sample during AMAT testing.

### 4.3. Characterizing the Strength-Function Relationship Based on AMAT Performance

Construct validity of the AMAT was also supported by the observed strength-function relationships. For example, the UE and LE QMA scores were more strongly associated with the functional AMAT subscale items than the total QMA score. Specificity of the composite regional strength scores moderately improved the observed strength-function relationships for nearly every task. Interestingly, LE QMA was strongly correlated with the sit-up task. However, a stronger correlation may have been attained with a specific measure of trunk strength, which was not included in this study. In addition, it is unclear why the total QMA score was more strongly correlated to the modified push-up task than was the UE QMA score. The muscle groups included in the composite UE QMA score did not include the horizontal adductors of the humerus, and the addition of this group may have improved this relationship. Our results also confirm the findings from other investigators regarding the positive relationship between task difficulty and strength [56]. Among the most difficult AMAT functional tasks were sit to stand and step-up (median score = 2.0). The highest strength-function correlations we observed involved tasks with a clear LE-bias ranging from 0.76 to 0.81. In contrast, the correlations for the UE-biased tasks ranged from 0.59 to 0.62. The large magnitude of association between muscle strength and LE-biased tasks observed in this study is similar to the findings of other studies of participants with neuromuscular disease [57].

### 4.4. Internal Consistency of the AMAT

While both AMAT subscales demonstrated good internal consistency, the functional subscale outperformed the endurance subscale. Frank muscle weakness can confound attempts to measure muscle endurance. Repeated or sustained tasks are designed to measure muscle endurance, but they also demand the requisite strength to attain the testing position. The distal weakness exhibited by the participants rendered the repeated heel raise test, an endurance AMAT subscale item, a* de facto* functional test contingent on strength. Therefore, severe neuromuscular disease that yields specific muscle groups with frank weakness would cause a series of muscle endurance tests to be divergent in their results, thus lowering the intercorrelation of the test items.

### 4.5. Utility of the AMAT: Cut Scores and Functional Performance Categories

The ability to derive meaning from the scores of a given outcome measure is a key arbiter of assessment tool utility. The determination of AMAT cut scores revealed significant categorical differences in physical performance. These observed differences included strength, walking, total ADL, ADL falling, and self-reported physical status. Participants categorized as having a “high” level of functional performance were at least twice as strong as those categorized as having a “low” level of functional performance. Similarly, walking distance was nearly three times farther in participants demonstrating a higher level of functional performance in comparison to people in the lowest functional category. This sharp contrast in physical functioning suggests that the AMAT cut scores may reveal clinically meaningful differences among the categorical groups. Clinicians may find that AMAT cut scores augment their ability to determine when additional rehabilitative interventions or more detailed assessments are indicated for patients with declining physical status. Moreover, AMAT cut scores may be used by researchers as part of the inclusion or exclusion criteria of a therapeutic trial, to aid group assignment based on the severity of physical impairment or provide a criterion for clinically meaningful improvement or worsening when participant AMAT scores shift in categorical rank. Despite the clear functional distinctions observed in the categorical grouping of our sample, additional study will be needed to better understand how the AMAT cut scores identified in this study apply to other samples and patient populations. Myopathy is a broad category of pathology that encompasses multiple neuromuscular disorders and myogenic diseases. Therefore, the AMAT was not created for the express purpose of assessing individuals with SBMA. Our preliminary data from previous and ongoing clinical studies suggest that the AMAT is a robust measure of physical performance in people with inclusion body myositis and that clinicians exhibit a high degree of reliability scoring AMAT performances by individuals with idiopathic inflammatory myopathies [17].

This performance-based test is intended for use by rehabilitation practitioners such as physicians, therapists, and nurses and may be conducted in physical therapy clinics, outpatient medical facilities, and rehabilitation units within a hospital setting. The emerging analytic properties of the AMAT, including the ability to monitor patient status over time and observe meaningful shifts in the AMAT functional level (i.e., low, moderate, and high), are valuable features of a test designed to characterize the physical performance of people with chronic degenerative conditions. Our findings in support of the construct validity and internal consistency of the AMAT complement our previous observations regarding the ability of the AMAT to assess disease progression. Fernández-Rhodes et al. [29] examined the efficacy and safety of dutasteride in characterizing disease progression over a 24-month period in the placebo-control SBMA group with a variety of secondary measures of impairment level and physical status. Motor unit number estimation, median compound muscle action potentials, and total QMA score detected an annual rate of decline from 1.6% to 2.3%. In contrast, the AMAT and the PCS score showed an annual decline of 4.5% and 5.2%, respectively. However, of these two measures, the AMAT was better at detecting a decline in physical status (*z* = 0.68, *P* = 0.004 versus *z* = 0.43, *P* = 0.054). Therefore, the AMAT may have utility in future clinical trials based on its favorable “signal-to-noise” ratio.

## 5. Limitations

Although the findings support the construct validity and internal consistency of the AMAT, this study had limitations. Our outcome measures did not include a direct measure of muscle endurance. While the capacity of muscles to exert sustained or repeated submaximal forces is consistent with the requirements of ADL performance and mobility, validation of the endurance AMAT subscale would have been improved by comparisons with an impairment-level measure of anaerobic endurance. The AMAT and other physical performance tests have important advantages over questionnaires regarding physical functioning. Nonetheless, questionnaires such as the ALSFRS-r incorporate important questions regarding bulbar muscle function and various nonmusculoskeletal features of ALS and SBMA that are not included in the AMAT. While the purpose and validity of the AMAT benefits from the integrity of its domains, other tests or questionnaires are required to address the consequences of neuromuscular disease that go beyond physical performance and mobility. Additionally, the cut scores used to categorize participants into AMAT functional levels in this study yielded statistically significant distinctions among the 3 subgroups. However, cut scores based on percentiles are dependent on the distribution of scores within a given sample. An alternative approach would be to use criterion-based cut scores derived from established markers of disablement. A successful implementation of this approach to cut scores and functional categories will require a larger sample size to allow for a sufficient allocation of people in each subgroup and ensure valid statistical comparisons. Finally, other analytic qualities, such as responsiveness, the minimal clinical important difference score, criterion validity of the endurance subscale, and discriminative validity using normative reference data, need to be explored to fully understand the clinical and research utility of the AMAT.

## 6. Conclusions

The AMAT is a standardized, performance-based tool that assesses functional limitations and muscle endurance in adults with myopathy. Our findings suggest that the AMAT has excellent construct validity and good internal consistency for adults with SBMA based on its significant associations with strength, objective and subjective physical performance measures, and self-reported physical status. The utility of the AMAT is further supported through the use of cut scores to characterize physical status based on low, moderate, or high levels of performance. These findings support the use of the AMAT as both a clinical assessment tool and outcome measure in future clinical trials of SBMA and merits further study in other adult-onset neuromuscular disease populations.

## Figures and Tables

**Table 1:** Physical performance assessments in patients with SBMA.

	SBMA mean ± SD (range)	Percentage of reference values^†^
Quantitative muscle assessment (kg)		
Upper extremity composite	66 ± 25 (18–140)	42%
Lower extremity composite	98 ± 41 (28–231)	65%
Total force	164 ± 63 (63–372)	55%
Adult Myopathy Assessment Tool		
Endurance score (range = 0–24, 24 = max score)	14.5 ± 5.3 (4–24)	60%
Functional score (range = 0–21, 21 = max score)	14.7 ± 5.4 (2–21)	70%
Total score (range = 0–45, 45 = max score)	29.2 ± 10.3 (9–45)	65%
Timed 2-minute walk (m)		
Distance walked	109 ± 50 (15–208)	51%

^†^Normative QMA values obtained from published reference values [22, 23]; AMAT results expressed as a percentage of the maximum attainable score; timed 2 minute walk results compared with published age and gender matched normal reference values [24].

Abbreviations: SBMA: spinal bulbar muscular atrophy; kg: kilograms; m: meters.

**Table 2:** AMAT item scores for patients with SBMA.

	Median score	IQR
Functional AMAT subscale items (range = 0–3)		
Supine to prone	3.0	2.0–3.0
Modified push-up	3.0	2.0–3.0
Sit-up	1.0	0.0–2.0
Supine to sit	3.0	2.0–3.0
Arm raise	3.0	2.0–3.0
Sit to stand	2.0	1.0–3.0
Step-up	2.0	1.0–3.0
Endurance AMAT subscale items (range = 0–4)		
Sustained head elevation	3.0	2.0–4.0
Repeated modified push-ups	1.0	0.0–2.0
Sustained arm raise	3.0	1.3–4.0
Sustained hip flexion	4.0	2.0–4.0
Sustained knee extension	4.0	4.0–4.0
Repeated heel raises	0.0	0.0–1.0

Abbreviations: SBMA: spinal bulbar muscular atrophy; IQR: interquartile range; AMAT: Adult Myopathy Assessment Tool.

**Table 3:** Pearson's correlation coefficients of the AMAT total score and SBMA outcome measures and phenotypic variables.

	AMAT Total Score	*P* value
Scaled total QMA	0.91	<0.0001
Timed 2-minute walk	0.85	<0.0001
ADL assessment	0.82	<0.0001
Physical component summary^§^	0.82	<0.0001
Total testosterone	0.62	<0.0001
Dihydrotestosterone	0.51	<0.0001
Free testosterone	0.49	0.0002
Age	−0.40	0.002
Disease duration^†^	−0.29	0.03
Mental component summary^§^	0.13	0.355

^§^Self-report of physical and mental status obtained from the physical component summary and mental component summary of the Medical Outcomes Study 36-item short form, version 2.

^†^Disease duration is defined as time from genetic diagnosis to study initial evaluation.

Abbreviations: SBMA: spinal bulbar muscular atrophy; AMAT: Adult Myopathy Assessment Tool; QMA: Quantitative Muscle Assessment; ADL: activities of daily living.

**Table 4:** Spearman's correlation coefficients of the scaled QMA strength values and AMAT Functional subscale items.

	Supine to prone	Push-up	Sit-up	Supine to sit	Arm raise	Sit to stand	Step-up
UE QMA	0.379	0.616	0.623	0.570	0.588	0.614	0.637
LE QMA	0.524	0.559	0.756	0.724	0.471	0.764	0.813

TOTAL QMA	0.487	0.628	0.745	0.687	0.553	0.739	0.777

Note: all *P* values are <0.001, except UE QMA and supine to prone, *P* = 0.004; all QMA values are scaled to body weight.

Abbreviations: UE: upper extremity; LE: lower extremity; AMAT: Adult Myopathy Assessment Tool; QMA: quantitative muscle assessment.

**Table 5:** AMAT cut scores. Use of AMAT cut scores to discriminate among low, moderate, and high levels of performance across several ICF domains of function.

AMAT			QMA		2MWT (m)		PCS		ADL
Functional level	Score	*N*	Mean (SD)						
1-low	0–24	19	2.48 (±0.70)		58.7 (±24.0)		27.3 (±7.3)		21.0 (±3.0)
2-moderate	25–34	18	3.27 (±0.77)		103.1 (±27.7)		31.5 (±8.3)		26.0 (±3.1)
3-high	35–45	19	5.48 (±1.26)		163.7 (±24.3)		43.9 (±9.81)		30.7 (±3.4)

ANOVA			*F* value (all *P* values, <0.001)						
			52.9		60.1		18.3		44.7

Tukey's HSD			*P* values						
1-2			0.02		0.003		0.341		<0.001
2-3			<0.001		<0.001		<0.001		<0.001
1–3			<0.001		<0.001		<0.001		<0.001

Note: Cut scores are based on the tertiles of the AMAT total score. QMA values have been scaled to body weight.

Abbreviations: AMAT: Adult Myopathy Assessment Tool; ICF: International Classification of Functioning; QMA: quantitative muscle assessment; 2MWT: timed 2 minute walk; (m) meters; PCS: Physical Component Summary (obtained from the Medical Outcomes Study 36-Item Short Form, Version 2); ADL: activities of daily living; (SD) standard deviation; ANOVA: analysis of variance; Tukey's HSD: Tukey's Honestly Significant Difference.

**Table 6:** The adult myopathy assessment tool (AMAT).

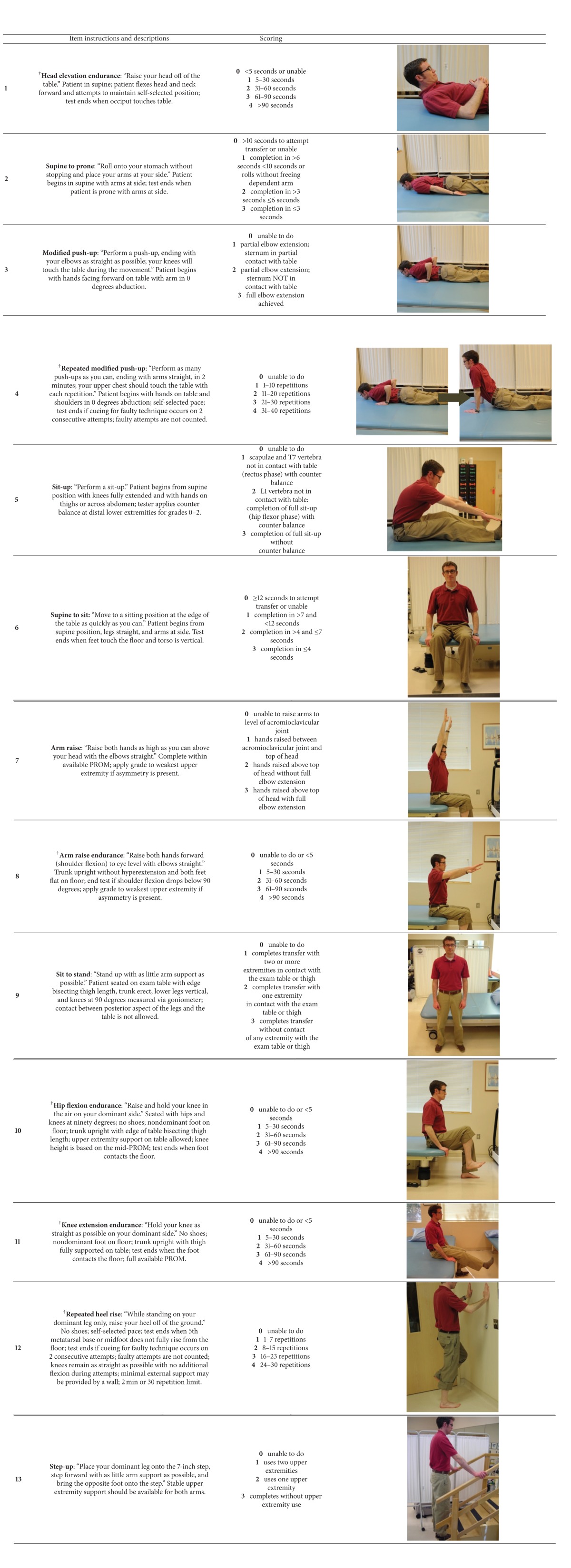

^†^Denotes AMAT endurance subscale items.

*General instructions:* all AMAT items should be performed in order 1 through 13 with at least one-minute rest period after each item. Rest periods exceeding one minute are dictated by the transition time required to set up proceeding AMAT tasks. Test ending criteria should be provided prior to each task attempt. Standby assistance is required for all items requiring upright mobility. Duration for all timed tasks should be recorded. *Required items*: stopwatch, examination table, goniometer or inclinometer, and stairs with a handrail.

*Scoring:* Each AMAT item is scored immediately after the task attempt is completed. The AMAT functional subscale (range = 0–21), AMAT endurance subscale (range = 0–24), and AMAT total score (range = 0–45) are calculated after test administration.

*Interpretation:* AMAT functional level (categorical ranks are based on the AMAT total score)

1-low 0–24

2-moderate 25–34

3-high 35–45

PROM: passive range of motion.

**Table 7:** Quantitative assessment of peak muscle force. The tested muscle groups, subject testing position, and orientation of the dynamometer strap are listed for the quantitative assessment of maximum isometric force^€^ using a fixed dynamometry load cell^*£*^.

Muscle group	Patient position	Strap position
Upper extremity		
Lateral pinch	Seated; elbow at 90°; midrange supination/pronation	None; pinch dynamometer
Hand grip	Seated; elbow at 90°; midrange supination/pronation	None; hand grip dynamometer
Wrist flexors	Seated; elbow at 90°; midrange supination/pronation	Ventral metacarpals with second stabilizing strap at dorsal proximal wrist
Elbow flexors	Supine; elbow at 90°; midrange supination/pronation	Radial distal forearm proximal to wrist
Elbow extensors	Supine; elbow at 90°; midrange supination/pronation	Ulnar distal forearm proximal to wrist
Shoulder abductors	Supine; shoulder and elbow at 90°	Lateral distal arm proximal to elbow
Lower extremity		
Ankle dorsiflexors	Supine; ankle at 90°	Around dorsal metatarsals
Knee extensors	Seated; hip and knee at 90°	Around ankle and proximal to malleolus
Hip flexors	Supine; hip and knee at 90°	Anterior distal femur and proximal to patella
Hip extensors	Supine; hip and knee at 90°	Posterior distal femur
Hip abductors	Seated; hip and knee at 90°	Lateral distal femur

^*€*^AEVERL Medical, LLC P.O. Box  170 Gainesville, GA 30503.

^*£*^Interface, 7401 East Butherus Drive, Scottsdale, AZ 85260.

## References

[1] Mänty M, de Leon CF, Rantanen T (2012). Mobility-related fatigue, walking speed, and muscle strength in older people. *The Journals of Gerontology A, Biological Sciences and Medical Sciences*.

[2] Rantanen T, Guralnik JM, Leveille S (1998). Racial differences in muscle strength in disabled older women. *Journals of Gerontology A, Biological Sciences and Medical Sciences*.

[3] Rantanen T (2003). Muscle strength, disability and mortality. *Scandinavian Journal of Medicine and Science in Sports*.

[4] Lowes LP, Alfano L, Viollet L (2012). Knee extensor strength exhibits potential to predict function in sporadic inclusion-body myositis. *Muscle and Nerve*.

[5] Jacobson BH, Smith D, Fronterhouse J, Kline C, Boolani A (2012). Assessment of the benefit of powered exercises for muscular endurance and functional capacity in elderly participants. *Journal of Physical Activity & Health*.

[6] Harris-Love MO (2003). Physical activity and disablement in the idiopathic inflammatory myopathies. *Current Opinion in Rheumatology*.

[7] Huber AM, Hicks JE, Lachenbruch PA (2001). Validation of the childhood health assessment questionnaire in the juvenile idiopathic myopathies. *Journal of Rheumatology*.

[8] Svantesson U, Österberg U, Thomeé R, Grimby G (1998). Muscle fatigue in a standing heel-rise test. *Scandinavian Journal of Rehabilitation Medicine*.

[9] Moxley RT (1994). Evaluation of neuromuscular function in inflammatory myopathy. *Rheumatic Disease Clinics of North America*.

[10] Josefson A, Romanus E, Carlsson J (1996). A functional index in myositis. *Journal of Rheumatology*.

[11] Guralnik JM (1997). Assessment of physical performance and disability in older persons. *Muscle and Nerve*.

[12] Brooke MH, Fenichel GM, Griggs RC (1983). Clinical investigation in Duchenne dystrophy: II. Determination of the “power” of therapeutic trials based on the natural history. *Muscle and Nerve*.

[13] McDonald CM, Abresch RT, Carter GT (1995). Profiles of neuromuscular diseases. Duchenne muscular dystrophy. *American Journal of Physical Medicine & Rehabilitation*.

[14] Brandt EN, Pope AM (1997). *Enabling America: Assessing the Role of Rehabilitation Science and Engineering*.

[15] World Health Organization (2001). *International Classification of Functioning, Disability and Health (ICF)*.

[16] McHorney CA, Tarlov AR (1995). Individual-patient monitoring in clinical practice: are available health status surveys adequate?. *Quality of Life Research*.

[17] Harris-Love MO, Joe G, Koziol DE (2004). Performance-based assessment of functional limitation and muscle endurance: reliability of the Adult Myositis Assessment Tool. *Journal of Neurologic Physical Therapy*.

[18] La Spada AR, Wilson EM, Lubahn DB, Harding AE, Fischbeck KH (1991). Androgen receptor gene mutations in X-linked spinal and bulbar muscular atrophy. *Nature*.

[19] Chahin N, Klein C, Mandrekar J, Sorenson E (2008). Natural history of spinal-bulbar muscular atrophy. *Neurology*.

[20] Ferrante MA, Wilbourn AJ (1997). The characteristic electrodiagnostic features of Kennedy’s disease. *Muscle Nerve*.

[21] Rhodes LE, Freeman BK, Auh S (2009). Clinical features of spinal and bulbar muscular atrophy. *Brain*.

[22] The National Isometric Muscle Strength (NIMS) Database Consortium (1996). Muscular weakness assessment: use of normal isometric strength data. *Archives of Physical Medicine and Rehabilitation*.

[23] Andrews AW, Thomas MW, Bohannon RW (1996). Normative values for isometric muscle force measurements obtained with hand-held dynamometers. *Physical Therapy*.

[24] Selman JPR, de Camargo AA, Santos J, Lanza FC, Dal Corso S (2014). Reference equation for the two-minute walk test in adults and the elderly. *Respiratory Care*.

[25] Banno H, Katsurio M, Suzuki K (2009). Phase 2 trial of leuprorelin in patients with spinal and bulbar muscular atrophy. *Annals of Neurology*.

[26] Goverover Y, O’Brien AR, Moore NB, DeLuca J (2010). Actual reality: a new approach to functional assessment in persons with multiple sclerosis. *Archives of Physical Medicine and Rehabilitation*.

[27] Shephard RJ (2003). Limits to the measurement of habitual physical activity by questionnaires. *British Journal of Sports Medicine*.

[28] van Weely SFE, van Denderen JC, Steultjens MPM (2012). Moving instead of asking? Performance-based tests and BASFI-questionnaire measure different aspects of physical function in ankylosing spondylitis. *Arthritis Research and Therapy*.

[29] Fernández-Rhodes LE, Kokkinis AD, White MJ (2011). Efficacy and safety of dutasteride in patients with spinal and bulbar muscular atrophy: a randomised placebo-controlled trial. *The Lancet Neurology*.

[30] Rossier P, Wade DT (2001). Validity and reliability comparison of 4 mobility measures in patients presenting with neurologic impairment. *Archives of Physical Medicine and Rehabilitation*.

[31] Stolwijk-Swüste JM, Beelen A, Lankhorst GJ (2008). SF36 physical functioning scale and 2-minute walk test advocated as core qualifiers to evaluate physical functioning in patients with late-onset sequelae of poliomyelitis. *Journal of Rehabilitation Medicine*.

[32] Light KE, Bebrman AL, Thigpen M, Triggs WJ (1997). The 2-minute walk test: a tool for evaluating walking endurance in clients with Parkinson’s disease. *Journal of Neurologic Physical Therapy*.

[33] Butland RJ, Pang J, Gross ER, Woodcock AA, Geddes DM (1982). Two-, six-, and 12-minute walking tests in respiratory disease. *British Medical Journal*.

[34] Subramony SH, May W, Lynch D (2005). Measuring Friedreich ataxia: Interrater reliability of a neurologic rating scale. *Neurology*.

[35] Lynch DR, Farmer JM, Tsou AY (2006). Measuring Friedreich ataxia: complementary features of examination and performance measures. *Neurology*.

[36] Riazi A, Hobart JC, Lamping DL (2003). Using the SF-36 measure to compare the health impact of multiple sclerosis and Parkinson’s disease with normal population health profiles. *Journal of Neurology Neurosurgery and Psychiatry*.

[37] Finas D, Bals-Pratsch M, Sandmann J (2006). Quality of life in elderly men with androgen deficiency. *Andrologia*.

[38] Ware JE, Kosinski M, Bjorner JB, Turner-Bowker DM, Gandek B, Maruish ME (2007). *User’s Manual for the SF-36v2 Health Survey*.

[39] Ware JE, Kosinski M, Dewey JE (2000). *How to Score Version Two of the SF-36—Health Survey*.

[40] Portney LG, Watkins MP (2009). *Foundations of Clinical Research: Applications to Practice*.

[41] Foster SL, Cone JD (1995). Validity issues in clinical assessment. *Psychological Assessment*.

[42] Munro BH (2001). *Statistical Methods for Health Care Research*.

[43] Field A (2009). *Discovering Statistics Using SPSS*.

[44] Jaric S (2003). Role of body size in the relation between muscle strength and movement performance. *Exercise and Sport Sciences Reviews*.

[45] Jaric S (2002). Muscle strength testing: use of normalisation for body size. *Sports Medicine*.

[46] Brown M (2008). Skeletal muscle and bone: effect of sex steroids and aging. *American Journal of Physiology—Advances in Physiology Education*.

[47] Rantanen T, Guralnik JM, Izmirlian G (1998). Association of muscle strength with maximum walking speed in disabled older women. *American Journal of Physical Medicine and Rehabilitation*.

[48] Doyu M, Sobue G, Mukai E (1992). Severity of X-linked recessive bulbospinal neuronopathy correlates with size of the tandem CAG repeat in androgen receptor gene. *Annals of Neurology*.

[49] La Spada AR, Roling DB, Harding AE (1992). Meiotic stability and genotype—phenotype correlation of the trinucleotide repeat in X-linked spinal and bulbar muscular atrophy. *Nature Genetics*.

[50] Atsuta N, Watanabe H, Ito M (2006). Natural history of spinal and bulbar muscular atrophy (SBMA): a study of 223 Japanese patients. *Brain*.

[51] Alexanderson H, Broman L, Tollbäck A, Josefson A, Lundberg IE, Stenström CH (2006). Functional Index-2: validity and reliability of a disease-specific measure of impairment in patients with polymyositis and dermatomyositis. *Arthritis Care and Research*.

[52] Lieber RL (2010). *Skeletal Muscle Structure, Function, and Plasticity: the Physiological Basis of Rehabilitation*.

[53] Harris-Love MO, Shrader JA, Davenport TE (2014). Are repeated single-limb heel raises and manual muscle testing associated with peak plantar-flexor force in people with inclusion body myositis?. *Physical Therapy*.

[54] Boyer JG, Ferrier A, Kothary R (2013). More than a bystander: the contributions of intrinsic skeletal muscle defects in motor neuron diseases. *Frontiers in Physiology*.

[55] Chahin N, Sorenson EJ (2009). Serum creatine kinase levels in spinobulbar muscular atrophy and amyotrophic lateral sclerosis. *Muscle and Nerve*.

[56] Salem GJ, Wang M-Y, Young JT, Marion M, Greendale GA (2000). Knee strength and lower- and higher-intensity functional performance in older adults. *Medicine and Science in Sports and Exercise*.

[57] Merlini L, Bertini E, Minetti C (2004). Motor function-muscle strength relationship in spinal muscular atrophy. *Muscle and Nerve*.

